# Advancements in extracellular vesicle therapy for neurodegenerative diseases

**DOI:** 10.37349/ent.2025.1004104

**Published:** 2025-05-06

**Authors:** Ningyun Hu, Liang Chen, Guoku Hu, Rong Ma

**Affiliations:** 1 Department of Pharmacology and Experimental Neuroscience, University of Nebraska Medical Center, Omaha, NE 68198-5880, USA; 2 Millard West High School, Omaha, NE 68135, USA; 3 Department of Computer Science and Technology, College of Mathematics and Computer, Shantou University, Shantou 515821, Guangdong, China; 4 Department of Pharmacology, School of Basic Medicine, Tongji Medical College, Huazhong University of Science and Technology, Wuhan 430030, Hubei, China

**Keywords:** Extracellular vesicles, exosomes, Alzheimer’s disease, Parkinson’s disease, Huntington’s disease, amyotrophic lateral sclerosis, multiple sclerosis

## Abstract

Neurodegenerative diseases represent a significant and growing challenge to public health worldwide. Current therapeutic strategies often fall short in halting or reversing disease progression, highlighting the urgent need for novel approaches. Extracellular vesicles (EVs) have garnered attention as potential therapeutic agents due to their role in intercellular communication and their ability to transport bioactive cargo, including proteins, nucleic acids, and lipids. This review provides a comprehensive overview of the biology of EVs, their involvement in neurodegenerative diseases, and the potential for EV-based therapies. We discuss the different types of EVs, their biogenesis, and their cargo composition, emphasizing their relevance to neurological processes such as protein misfolding, neuroinflammation, and oxidative stress. Preclinical studies investigating EVs as carriers of therapeutic cargo and their ability to promote neuronal survival and regeneration are examined, with a focus on evidence from animal models of neurodegenerative disorders. We explore the use of EVs in the treatment of neurodegenerative diseases, including ongoing clinical trials, methods for EV isolation and modification, and future perspectives on personalized EV-based therapies designed to meet the unique needs of individual patients. Overall, this review highlights the potential of EVs as a promising avenue for neurodegenerative disease therapy, while also addressing key research gaps and translational hurdles that need to be overcome for their successful clinical implementation.

## Graphical abstract.

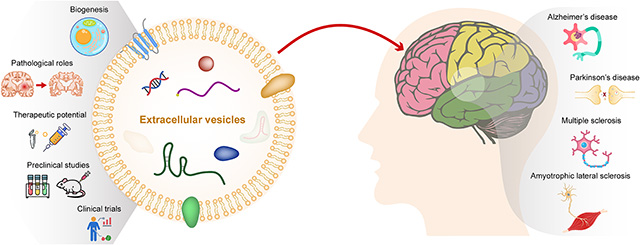

**EVs are a promising avenue for neurodegenerative disease therapy.** Some icons are designed by Freepik (http://www.freepik.com/). Icon made by dDara from www.flaticon.com; Icon made by Freepik from www.flaticon.com; Icon made by AomAm from www.flaticon.com; Icon made by Flat Icons from www.flaticon.com; Icon made by Graficon from www.flaticon.com; Icon made by Parzival’ 1997 from www.flaticon.com; Icon made by Leremy from www.flaticon.com

## Introduction

Neurodegenerative diseases encompass a wide array of conditions distinguished by progressive dysfunction, loss of neurons, and inflammation in the central nervous system (CNS) or peripheral nervous system (PNS). These conditions have significant implications for public health, affecting millions of individuals worldwide and imposing substantial economic burdens on healthcare systems. Alzheimer’s disease (AD), Parkinson’s disease (PD), Huntington’s disease (HD), amyotrophic lateral sclerosis (ALS), and multiple sclerosis (MS) are the most common neurodegenerative diseases.

### Overview of neurodegenerative diseases

AD is the leading cause of dementia, characterized by the accumulation of amyloid-beta (Aβ) plaques and tau protein tangles in the brain, which progressively result in cognitive decline and memory loss [1]. PD manifests through the depletion of dopamine-producing neurons within the substantia nigra area of the brain, leading to motor manifestations like tremors, rigidity, and bradykinesia [2]. HD is a hereditary disorder caused by a mutation in the huntingtin gene, leading to progressive motor dysfunction, cognitive decline, and psychiatric symptoms [3]. ALS represents a motor neuron disorder marked by the deterioration of both upper and lower motor neurons, resulting in muscle weakness, paralysis, and respiratory failure [4]. MS is an autoimmune condition impacting the CNS, leading to inflammation, demyelination, and neurodegeneration, resulting in a wide range of neurological symptoms [5]. These neurodegenerative diseases share common features such as protein misfolding, neuroinflammation, oxidative stress, mitochondrial dysfunction, and synaptic loss, contributing to progressive neuronal damage and functional impairment.

### Current challenges in therapy development

Despite extensive research efforts and advances in understanding the molecular mechanisms underlying neurodegenerative diseases, developing effective therapies remains a daunting challenge. Several key challenges impede progress in therapy development: 1) Complexity of neurological processes: Neurodegenerative diseases are multifactorial and heterogeneous, involving intricate interactions between genetic, environmental, and lifestyle factors. Targeting multiple pathological pathways simultaneously presents a formidable challenge. 2) Limited disease-modifying treatments: Most available treatments focus on managing symptoms and providing temporary relief rather than halting or reversing disease progression. There is a critical need for disease-modifying therapies that can slow or stop neurodegeneration. 3) Blood-brain barrier (BBB) impediments: The BBB restricts the passage of therapeutics and limits their access to the CNS, posing a significant barrier to drug delivery and efficacy in neurodegenerative disorders. 4) Early diagnosis and biomarkers: Lack of reliable biomarkers for early disease detection and monitoring disease progression hinders timely intervention and personalized treatment strategies.

### Rationale for exploring extracellular vesicles as a therapeutic approach

In recent years, extracellular vesicles (EVs) have emerged as promising candidates for neurodegenerative disease therapy due to their unique properties and functions [6]. EVs are membrane-bound vesicles released by various cell types, including neurons, glial cells, and immune cells, into the extracellular environment [7, 8]. EVs play crucial roles in cell-to-cell communication by transferring bioactive molecules, including proteins, lipids, nucleic acids (such as miRNAs), and signaling molecules [9]. The rationale for exploring EVs as a therapeutic approach stems from several key factors: 1) Intercellular communication: EVs serve as vehicles for intercellular communication, allowing the transfer of biomolecules between cells in a paracrine or endocrine manner. This communication network plays a vital role in maintaining tissue homeostasis and regulating physiological processes [10]. 2) Cargo delivery: EVs encapsulate a diverse cargo of biomolecules, including growth factors, neurotrophic factors, antioxidants, anti-inflammatory agents, and genetic material. This cargo can modulate cellular functions, promote cell survival, repair damaged tissues, and regulate immune responses [11, 12]. 3) Crossing biological barriers: EVs can traverse biological barriers, including the BBB, enabling targeted delivery of therapeutic cargo to the CNS. This property is crucial for accessing the brain and spinal cord, which are often challenging to target with conventional drug delivery methods [13, 14]. 4) Natural biocompatibility: EVs are derived from cells and exhibit natural biocompatibility, reducing the risk of immune rejection or adverse reactions compared to synthetic drug delivery systems. They can also be engineered or modified to enhance their therapeutic properties and targeting specificity [15, 16]. 5) Disease-modifying potential: Preclinical studies have demonstrated the disease-modifying potential of EV-based therapies in various neurodegenerative disease models. EVs derived from regulatory T cells and stem cells, such as mesenchymal stem cells (MSCs) and neural progenitor cells (NPCs), show neuroprotective, anti-inflammatory, antioxidant, and regenerative effects in vitro and in vivo [17, 18]. 6) Clinical translation: EV-based therapies have advanced into clinical trials for neurodegenerative diseases, demonstrating safety, feasibility, and preliminary efficacy in early-phase studies [19]. These trials pave the way for further exploration of EVs as a viable therapeutic strategy in clinical settings.

Overall, the unique properties of EVs make them attractive candidates for developing innovative and targeted therapies for neurodegenerative diseases. Understanding the biology of EVs, including their roles in intercellular communication, cargo composition, delivery mechanisms, and therapeutic potential, is essential for harnessing their benefits in combating neurodegeneration and improving patient outcomes. Continued research efforts and collaborative initiatives are needed to overcome the challenges in EV-based therapy development and facilitate their clinical translation into effective treatments for neurodegenerative disorders.

## Biology of EVs

### Types of EVs

EVs are a heterogeneous group of membrane-bound vesicles released by cells into the extracellular environment and play essential roles in intercellular communication, carrying bioactive molecules that can modulate cellular functions and responses. EVs can be broadly classified into three main types based on their biogenesis and size: exosomes, microvesicles (also known as ectosomes or shedding vesicles), and apoptotic bodies.

#### Exosomes

Exosomes are small EVs (30–150 nm in diameter) originating from the endosomal pathway [20]. Exosomes are formed through a series of intracellular processes involving the endosomal sorting complex required for transport (ESCRT) machinery and lipid raft-dependent mechanisms [8]. Exosomes are typically released from cells upon the fusion of multivesicular bodies (MVBs) with the plasma membrane. Exosomes contain a diverse cargo of proteins, lipids, nucleic acids (including miRNAs, mRNAs, and long non-coding RNAs), and signaling molecules [21]. Exosomes are involved in cell-to-cell communication, immune regulation, tissue repair, and disease pathogenesis [22–24].

#### Microvesicles

Microvesicles are larger (100–1,000 nm in diameter) EVs that bud directly from the plasma membrane [25]. Microvesicles are generated by outward budding and fission of the plasma membrane, leading to the release of vesicles into the extracellular space [26]. Microvesicles carry a similar cargo as exosomes, including proteins, lipids, and nucleic acids. They play roles in cell signaling, coagulation, inflammation, angiogenesis, and cancer progression. Microvesicles are distinct from exosomes in their biogenesis pathway and size range, although there can be some overlap in cargo composition between these two EV types [27].

#### Apoptotic bodies

Apoptotic bodies are larger EVs (1–5 μm in diameter) released by cells undergoing programmed cell death (apoptosis) [28]. During apoptosis, the cell undergoes structural changes, condenses its contents, and forms apoptotic bodies containing cellular organelles, DNA fragments, and cytoplasmic components. Apoptotic bodies are phagocytosed by neighboring cells or macrophages, contributing to the clearance of dying cells and maintaining tissue homeostasis. While apoptotic bodies are not typically considered traditional EVs involved in intercellular communication, they are important in the context of cell death and immune regulation.

### Biogenesis and release mechanisms

**Figure 1.**
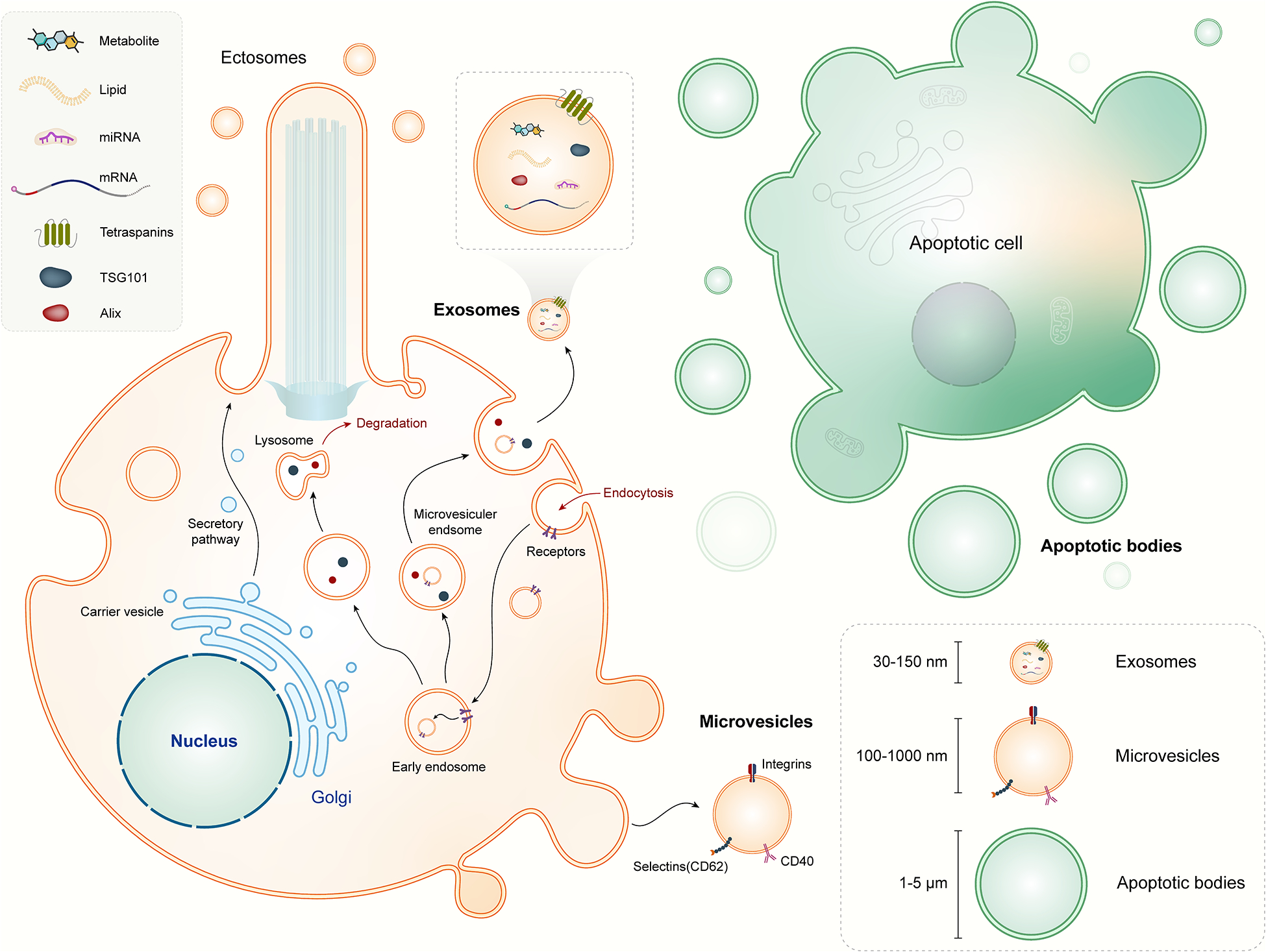
Types and biogenesis of extracellular vesicles. Extracellular vesicles (EVs) can be broadly categorized into three main types: (a) Exosomes originate within the endosomal network and are released upon fusion of multi-vesicular bodies with the plasma membrane. Exosomes typically range in size from 30 to 150 nm; (b) Microvesicles/microparticles/ectosomes formed through outward budding and fission of the plasma membrane or ciliary membrane. Microvesicles typically measure between 100 and 1,000 nm in size; and (c) Apoptotic bodies exhibit heterogeneity in size ranging from 1 to 5 μm. These apoptotic bodies are released as blebs from cells undergoing apoptosis. Icon made by Freepik from www.flaticon.com

The biogenesis of EVs involves complex cellular processes that vary depending on the type of EV and the cellular context (Figure 1). Here, we discuss the general mechanisms of EV biogenesis and release: 1) Exosome biogenesis: Exosomes originate from the endosomal pathway, starting with the formation of early endosomes from the inward budding of the plasma membrane. These early endosomes mature into late endosomes or MVBs through a process involving the ESCRT proteins [20]. The ESCRT machinery facilitates the sorting of cargo molecules into intraluminal vesicles (ILVs) within MVBs. Subsequently, MVBs can either fuse with lysosomes for cargo degradation or fuse with the plasma membrane for exosome release into the extracellular space. ESCRT-independent mechanisms, such as ceramide-mediated budding and tetraspanin-enriched microdomains, also contribute to exosome biogenesis. 2) Microvesicle formation: Microvesicles are formed by outward budding and shedding of the plasma membrane. This process involves cytoskeletal remodeling, membrane lipid rearrangements, and membrane curvature generation. Specific proteins, such as ADP-ribosylation factor 6 (ARF6), phospholipase D (PLD), and ESCRT components, participate in microvesicle biogenesis. Upon budding, microvesicles are released into the extracellular milieu, where they can interact with neighboring cells or distant targets [29]. Ciliary ectosomes, or ciliary microvesicles, are a specialized type of microvesicles that are released from the cilia of cells [30]. Cilia are small, finger-like projections found on the surface of many cell types, and they play essential roles in cell signaling, movement, and sensory perception [31, 32]. When cilia release ectosomes, these vesicles can carry specific cargo that reflects the unique composition and function of ciliary membranes [33]. The ectosome cargo often includes ciliary membrane proteins, signaling molecules, and other biomolecules involved in cilia-related processes. 3) Apoptotic body release: Apoptotic bodies are generated during programmed cell death (apoptosis), a highly regulated process involving caspase activation, DNA fragmentation, and cytoplasmic condensation [34]. As cells undergo apoptosis, they form apoptotic bodies containing cellular debris, organelles, and fragmented DNA. These apoptotic bodies are then released into the extracellular space through membrane blebbing and vesiculation. Phosphatidylserine exposure on the outer membrane of apoptotic bodies facilitates their recognition and phagocytosis by macrophages or neighboring cells, promoting the clearance of dying cells and maintaining tissue integrity [35–37]. Additionally, autophagy is a critical process in the biogenesis of EVs, including microvesicles, exosomes, and apoptotic bodies [38–40]. While autophagy is primarily known for degrading cellular components, it also facilitates the packaging of cellular debris, misfolded proteins, and nucleic acids into EVs. Thus, EVs serve as carriers of bioactive molecules, playing significant roles in intercellular communication and disease processes. Furthermore, lipid rafts, and cholesterol- and sphingolipid-enriched microdomains within the plasma membrane are essential for EV formation [41, 42]. They act as organizational platforms for signaling molecules and mediate the selective sorting of cargo, ultimately shaping the composition, function, and biological activity of EVs. This interplay between autophagy and lipid raft-mediated mechanisms highlights the complexity of EV biogenesis and their functional diversity in physiological and pathological contexts.

### Composition of EV cargo

EVs carry diverse biomolecules, encompassing proteins, nucleic acids, lipids, and metabolites. The composition of EV cargo reflects the cellular origin, physiological state, and environmental cues. Here, we highlight the major components of EV cargo: 1) Proteins: EVs contain a wide range of proteins derived from the parent cells, including membrane proteins, cytosolic proteins, and signaling molecules. These proteins play diverse roles in cell signaling, immune modulation, cell adhesion, and tissue repair. EVs are enriched in specific protein families, such as tetraspanins (CD9, CD63, CD81), heat shock proteins (HSP70, HSP90), integrins, major histocompatibility complex (MHC) molecules, and cytoskeletal proteins [43]. The protein cargo of EVs can vary depending on the cellular context, activation state, and cargo sorting mechanisms. Autoantigens play a crucial role in the pathogenesis of autoimmune diseases. EVs can carry autoantigens to immune cells, including dendritic cells, B cells, and T cells, stimulating immune responses and driving the production of autoantibodies [24, 44]. This process can amplify inflammatory pathways and contribute to the breakdown of immune tolerance. The active involvement of EVs in autoimmunity highlights their significance as key mediators of intercellular communication and underscores their potential as therapeutic targets for addressing immune dysregulation, which is often observed in neurodegenerative diseases. 2) Nucleic acids: EVs contain various nucleic acids, including mRNAs, miRNAs, long non-coding RNAs, transfer RNAs (tRNAs), and genomic DNA fragments. These nucleic acids are encapsulated within EVs and protected from degradation, allowing them to be transferred between cells and modulate gene expression. miRNAs carried by EVs can regulate target gene expression post-transcriptionally, influencing cellular processes such as proliferation, apoptosis, inflammation, and differentiation. EV-mediated transfer of nucleic acids contributes to intercellular communication, genetic exchange, and functional modulation of recipient cells [8]. 3) Lipids: EVs contain a lipid bilayer membrane derived from the parent cell plasma membrane or endosomal compartments. This lipid membrane encapsulates the cargo molecules within EVs and provides stability and protection during vesicle trafficking and uptake. Lipid composition analysis of EVs reveals enrichment in specific lipid species, including phospholipids, cholesterol, sphingolipids, and glycerophospholipids. Lipid rafts and microdomains within EV membranes play roles in cargo sorting, vesicle budding, and cellular uptake processes. Lipid molecules carried by EVs can modulate membrane dynamics, cell signaling pathways, and cellular responses in recipient cells [45]. 4) Metabolites and small molecules: EVs can also transport metabolites, small molecules, and bioactive compounds derived from cellular metabolism. These include amino acids, sugars, nucleotides, vitamins, enzymes, neurotransmitters, and secondary messengers. The presence of metabolites in EV cargo reflects the metabolic activity, nutrient status, and functional state of the parent cells [46, 47]. EV-mediated transfer of metabolites can regulate metabolic pathways, energy metabolism, cellular signaling, and physiological responses in recipient cells, influencing cellular functions and homeostasis.

Overall, the composition of EV cargo is diverse and dynamic, reflecting the complex interplay of cellular processes, environmental cues, and physiological conditions. The cargo molecules carried by EVs contribute to their functional effects on recipient cells and tissues, influencing cellular signaling, gene expression, metabolic pathways, immune responses, and tissue homeostasis. Understanding the composition and functional significance of EV cargo is crucial for elucidating the roles of EVs in intercellular communication, disease pathogenesis, and therapeutic applications. Continued research efforts are needed to characterize the cargo diversity, regulatory mechanisms, and functional implications of EV-mediated cargo transfer in health and disease.

## Role of EVs in neurodegenerative diseases

### EV involvement in intercellular communication

EVs function as conveyors of bioactive molecules, enabling them to transmit information among cells and regulate cellular activities. In the context of neurodegenerative diseases, EV-mediated communication is implicated in disease pathogenesis, progression, and potential therapeutic interventions [48, 49]. 1) Cell-to-cell signaling: EVs serve as vehicles for cell-to-cell signaling, enabling the transfer of signaling molecules, growth factors, cytokines, and neurotransmitters between neurons, glial cells, and immune cells. This communication network regulates neuronal survival, synaptic plasticity, neurogenesis, and immune responses in the CNS [50, 51]. Dysregulation of EV-mediated signaling pathways can contribute to neurodegenerative processes and neuronal dysfunction [11, 22]. 2) Neuronal network modulation: EVs participate in the modulation of neuronal networks by influencing synaptic transmission, neuronal excitability, and synaptic plasticity. EVs can carry synaptic proteins, neurotransmitters, and synaptic vesicle components, affecting synaptic function and connectivity. EV-mediated communication between neurons and glial cells regulates neuronal homeostasis, neuronal support, and responses to neuronal injury and stress [52, 53]. 3) Immune regulation: EVs derived from immune cells, such as microglia, astrocytes, and peripheral immune cells, play roles in immune regulation and neuroinflammation. EVs carry immunomodulatory factors, cytokines, chemokines, and antigens that regulate immune cell activation, polarization, migration, and interactions with neurons and glial cells. EV-mediated immune responses in neurodegenerative diseases can have both protective and detrimental effects, depending on the context and balance of pro-inflammatory and anti-inflammatory signals [54–56]. 4) Neurotrophic support: EVs derived from neural stem cells, MSCs, and glial cells can provide neurotrophic support, promote neuronal survival, and enhance neurodegeneration. EVs carry growth factors (e.g., brain-derived neurotrophic factor, nerve growth factor), neurotrophic factors, and extracellular matrix components that support neuronal growth, axonal regeneration, and synaptogenesis. EV-mediated neurotrophic effects contribute to neuroprotective mechanisms and tissue repair in neurodegenerative conditions [57–59].

Overall, EVs are key players in intercellular communication within the nervous system, regulating neuronal functions, immune responses, and tissue homeostasis. Understanding the roles of EV-mediated communication in neurodegenerative diseases is essential for elucidating disease mechanisms and developing targeted therapeutic strategies.

### EV-mediated transfer of pathological proteins (e.g., tau, alpha-synuclein)

Neurodegenerative diseases are characterized by the accumulation and spread of pathological proteins, such as tau, alpha-synuclein, Aβ, and huntingtin, in the CNS [60–63]. EVs have been implicated in the intercellular transfer and propagation of these pathological proteins, contributing to disease progression and neuronal dysfunction. The transfer of pathological proteins via EVs has significant implications for disease pathogenesis, protein aggregation, neurotoxicity, and potential therapeutic interventions. 1) Tau protein propagation: Tau protein is a microtubule-associated protein that undergoes abnormal hyperphosphorylation and aggregation in neurodegenerative diseases such as AD and frontotemporal dementia (FTD) [64]. EVs released from neurons and glial cells can carry pathological forms of tau, including hyperphosphorylated tau and tau oligomers [65]. EV-associated tau species can be transferred between cells, promoting the seeding and spread of tau pathology in the brain. EV-mediated tau propagation contributes to neuronal dysfunction, synaptic impairment, and cognitive decline in tauopathies [66–68]. 2) Alpha-synuclein spreading: Alpha-synuclein is a presynaptic protein implicated in PD and related synucleinopathies. Abnormal aggregation of alpha-synuclein leads to the formation of Lewy bodies and Lewy neurites, contributing to dopaminergic neuron degeneration and motor symptoms in PD [69, 70]. EVs released from neurons and glial cells can transport alpha-synuclein aggregates, oligomers, and fibrils, facilitating the intercellular spread of alpha-synuclein pathology [71, 72]. EV-mediated alpha-synuclein spreading contributes to neuronal toxicity, synapse dysfunction, and neuroinflammation in synucleinopathies [73]. 3) Prion-like mechanisms: The transfer of pathological proteins via EVs exhibits prion-like properties, involving templated protein misfolding and seeding of protein aggregates in recipient cells [74, 75]. EV-associated pathological proteins can induce conformational changes, nucleate protein aggregation, and propagate neurotoxicity in a prion-like manner [76, 77]. This prion-like spreading mechanism contributes to the progressive nature of neurodegenerative diseases and the amplification of protein pathology across brain regions. 4) EV cargo sorting and regulation: The sorting of pathological proteins into EVs involves specific mechanisms, including protein-protein interactions, post-translational modifications, and cargo sorting machinery [78, 79]. Cells regulate the packaging and release of pathological proteins into EVs, influencing the composition and neurotoxicity of EV cargo [80]. Modulating EV cargo sorting pathways and protein quality control mechanisms may represent therapeutic targets for inhibiting the propagation of pathological proteins in neurodegenerative diseases.

Understanding the mechanisms underlying EV-mediated transfer of pathological proteins is critical for developing strategies to block protein spreading, disrupt prion-like propagation, and mitigate neurotoxicity in neurodegenerative disorders. Targeting EV-mediated protein transfer pathways may offer novel therapeutic approaches for slowing disease progression and preserving neuronal function.

### Impact of EVs on neuroinflammation and oxidative stress

Neuroinflammation and oxidative stress are hallmark features of neurodegenerative diseases, contributing to neuronal damage, synaptic dysfunction, and disease progression [81–83]. EVs play diverse roles in modulating neuroinflammatory responses, oxidative stress pathways, and immune-mediated mechanisms in the CNS [84–86]. Understanding the impact of EVs on neuroinflammation and oxidative stress is crucial for elucidating disease mechanisms and developing targeted therapeutic interventions. 1) Immunomodulatory effects: EVs derived from immune cells, glial cells, and neurons can modulate immune responses and neuroinflammatory processes in the CNS [54, 87]. EVs carry immunomodulatory factors, cytokines, chemokines, and miRNAs that regulate immune cell activation, polarization, migration, and cytokine production [18, 88]. EV-mediated communication between immune cells and neural cells influences neuroinflammatory signaling pathways, microglial activation states, astrocyte reactivity, and immune cell infiltration into the brain. Dysregulation of EV-mediated immunomodulation contributes to neuroinflammatory cascades, neurotoxicity, and neuronal damage in neurodegenerative diseases. 2) Oxidative stress regulation: EVs regulate oxidative stress responses and redox signaling pathways in neuronal and glial cells, as EVs carry antioxidant enzymes [e.g., superoxide dismutase (SOD), catalase], redox regulators, and molecules involved in cellular antioxidant defense mechanisms. EV-mediated transfer of antioxidant molecules and signaling factors modulates oxidative stress pathways, mitochondrial function, and cellular redox balance [89–91]. EVs released in response to oxidative stress can also contain damage-associated molecular patterns (DAMPs), reactive oxygen species (ROS), and oxidative stress-related proteins, contributing to oxidative damage, cellular senescence, and neurodegeneration [92]. The balance between oxidative stress and antioxidant responses mediated by EVs influences neuronal survival, synaptic integrity, and disease progression in neurodegenerative conditions [90]. 3) BBB dysfunction: EVs can influence BBB integrity, permeability, and neurovascular interactions in neurodegenerative diseases. EVs derived from endothelial cells, pericytes, astrocytes, and immune cells carry molecules involved in BBB regulation, vascular homeostasis, and endothelial function [93–97]. EV-mediated transfer of BBB-modulating factors, inflammatory mediators, and miRNAs can disrupt BBB integrity, promote endothelial dysfunction, and enhance neuroinflammatory responses. BBB dysfunction mediated by EVs contributes to neuroinflammation, neurovascular pathology, and neuronal damage in neurodegenerative disorders. 4) Neuroprotective effects: Despite their pro-inflammatory roles, EVs can also exert neuroprotective effects and promote neuronal survival in neurodegenerative diseases. EVs derived from stem cells, NPCs, and glial cells carry neurotrophic factors, growth factors, anti-inflammatory molecules, and antioxidant enzymes that support neuronal viability, axonal regeneration, and synaptic plasticity [98–101]. EV-mediated neuroprotection involves the modulation of cellular stress responses, trophic support mechanisms, and tissue repair processes. Harnessing the neuroprotective potential of EVs may offer therapeutic strategies for mitigating neuroinflammation, oxidative stress, and neurodegeneration in CNS disorders. 5) Therapeutic potential: EVs represent promising therapeutic candidates for modulating neuroinflammation and oxidative stress in neurodegenerative diseases. Engineered EVs and EV-derived nanoparticles can be designed to deliver therapeutic cargo, anti-inflammatory agents, antioxidants, and neuroprotective molecules to target cells in the CNS. Strategies for enhancing the therapeutic efficacy, targeting specificity, and bioavailability of EV-based therapies are under investigation for neurodegenerative disorders. EV-based interventions aimed at modulating neuroinflammation, oxidative stress, and immune responses hold the potential for slowing disease progression, preserving neuronal function, and improving clinical outcomes [48].

In summary, EVs play multifaceted roles in neuroinflammation, oxidative stress, immune regulation, and neuroprotection in neurodegenerative diseases. Their impact on neuronal homeostasis, glial activation, BBB function, and cellular responses contributes to disease pathogenesis and potential therapeutic interventions. Further research is needed to elucidate the mechanisms underlying EV-mediated effects on neuroinflammation and oxidative stress, optimize EV-based therapeutic strategies, and translate these findings into clinical applications for neurodegenerative disorders.

## Preclinical studies utilizing EVs

EVs have garnered significant interest as potential therapeutic tools for neurodegenerative diseases due to their ability to transfer bioactive molecules and modulate cellular functions [18]. Preclinical studies have explored various aspects of EV-based therapies, including their role as carriers of therapeutic cargo, strategies for promoting neuronal survival and regeneration, and evidence from animal models of neurodegeneration [15, 102, 103].

### EVs as carriers of therapeutic cargo (RNA, small molecules, etc.)

**Figure 2.**
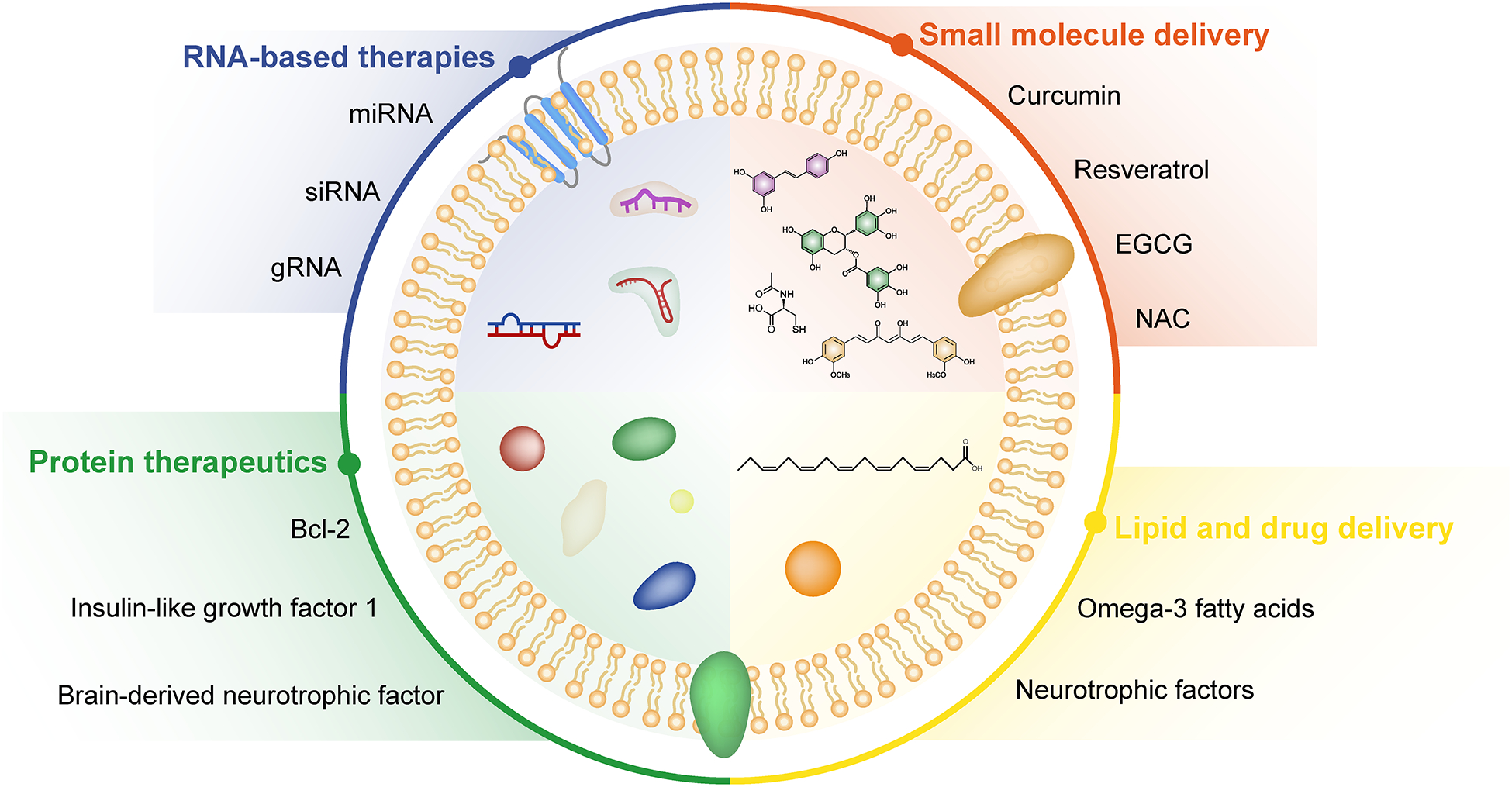
Extracellular vesicles as carriers of therapeutic cargo. Extracellular vesicles (EVs) are capable of transporting and delivering a diverse range of therapeutic agents, including RNA-based therapies, small molecule drugs, protein therapeutics, and lipid-based treatments. EGCG: epigallocatechin gallate; NAC: *N*-acetylcysteine

One of the key advantages of EVs is their ability to encapsulate and deliver therapeutic cargo to target cells within the CNS [13, 23, 104–106]. Preclinical studies have demonstrated the potential of EVs as carriers of various types of therapeutic molecules, including RNA (such as miRNAs, mRNAs, and siRNAs), small molecules, proteins, and lipids (Figure 2). These studies have highlighted the efficacy of EV-mediated delivery in modulating cellular pathways, gene expression, and disease progression in neurodegenerative disorders. 1) RNA-based therapies: EVs can transport different forms of RNA, including miRNAs that regulate gene expression post-transcriptionally [8, 107]. Preclinical studies have shown that EVs loaded with specific miRNAs can modulate neuronal survival, synaptic plasticity, inflammation, and neuroprotection in neurodegenerative diseases. For example, EVs carrying miR-124a, miR-29b, or miR-133b have been investigated for their neuroprotective effects in AD, PD, and HD models [108–112]. EV-based RNA therapies hold promise for targeted interventions and disease-modifying strategies. 2) Small molecule delivery: EVs can also transport small molecules, such as neuroprotective compounds, antioxidants, anti-inflammatory agents, and signaling modulators [113, 114]. Preclinical studies have explored the use of EVs loaded with small molecules to mitigate oxidative stress, reduce neuroinflammation, promote neurogenesis, and enhance synaptic function in neurodegenerative conditions. Examples include EVs loaded with curcumin, resveratrol, epigallocatechin gallate (EGCG), or *N*-acetylcysteine (NAC) for neuroprotection and disease-modifying effects in AD, PD, and ALS models [115–119]. 3) Protein therapeutics: EVs have the capacity to carry proteins, peptides, growth factors, and enzymes that exert neuroprotective, neurotrophic, or regenerative effects. Preclinical studies have investigated EV-mediated delivery of neurotrophins (e.g., brain-derived neurotrophic factor, nerve growth factor), growth factors (e.g., insulin-like growth factor 1, fibroblast growth factor), and anti-apoptotic proteins (e.g., Bcl-2, XIAP) for promoting neuronal survival, axonal regeneration, and functional recovery in neurodegenerative diseases [120, 121]. EV-based protein therapeutics offer targeted delivery and sustained release kinetics, enhancing their therapeutic potential.

Overall, preclinical studies utilizing EVs as carriers of therapeutic cargo have demonstrated the feasibility, efficacy, and versatility of EV-based delivery systems for neurodegenerative diseases. These studies provide insights into the mechanisms of EV-mediated cargo transfer, biodistribution, cellular uptake, and therapeutic effects in animal models, laying the foundation for translational research and clinical applications.

### Evidence from animal models of neurodegeneration

Animal models play a critical role in preclinical research to investigate the therapeutic potential of EVs in neurodegenerative diseases. These models recapitulate key aspects of disease pathology, molecular mechanisms, and behavioral phenotypes, allowing researchers to assess the efficacy, safety, and translational relevance of EV-based therapies (Table 1). Evidence from animal models provides valuable insights into the therapeutic effects of EVs on neuronal function, disease progression, and neuroprotective mechanisms. 1) AD models: Animal models of AD, such as transgenic mice expressing amyloid precursor protein (APP) and presenilin mutations, have been used to study the therapeutic effects of EVs on Aβ pathology, tau pathology, synaptic dysfunction, and cognitive impairment. EVs carrying medicine, miRNAs targeting tau pathology, or neurotrophic factors have shown neuroprotective effects, reduced amyloid deposition, improved synaptic plasticity, and cognitive enhancement in AD models [122, 123]. EV-based therapies targeting Aβ clearance, tau phosphorylation, and neuroinflammatory pathways hold promise for AD treatment [123–128]. 2) PD models: Animal models of PD, including toxin-induced models (e.g., MPTP, rotenone) and genetic models (e.g., alpha-synuclein transgenic mice), have been utilized to investigate EV-mediated neuroprotection, dopaminergic neuron survival, motor function, and mitochondrial dynamics. EVs carrying neurotrophic factors, antioxidants, mitochondrial enhancers, or alpha-synuclein-targeting molecules have shown therapeutic effects, reduced neuroinflammation, and improved motor behavior in PD models [129–132]. EV-based strategies for promoting dopaminergic neuron viability, mitochondrial function, and neuroregeneration are under investigation for PD therapy. 3) HD models: Animal models of HD, such as transgenic mice expressing mutant huntingtin protein (mHTT), have been employed to study EV-based therapies targeting mHTT aggregation, oxidative stress, synaptic dysfunction, and neuronal degeneration. EVs carrying miRNAs targeting mHTT expression, neuroprotective factors, or gene editing tools have shown potential for reducing mHTT levels, improving neuronal survival, and ameliorating motor deficits in HD models [133–136]. EV-mediated delivery of disease-modifying agents, neurotrophic factors, and RNA-based therapeutics holds promise for HD treatment strategies. 4) ALS models: Animal models of ALS, including transgenic mice expressing mutant SOD1 or TDP-43 mutations, have been utilized to investigate EV-mediated neuroprotection, motor neuron survival, neuromuscular function, and disease progression. EVs carrying anti-inflammatory molecules, growth factors, mitochondrial enhancers, or RNA-based therapies have shown therapeutic effects, reduced neuroinflammation, and improved motor performance in ALS models [137–140]. EV-based strategies for promoting motor neuron viability, axonal integrity, and neuroregeneration are being explored for ALS therapy. 5) MS models: Animal models of MS, such as experimental autoimmune encephalomyelitis (EAE) in rodents, have been used to study EV-mediated immunomodulation, neuroprotection, remyelination, and functional recovery. EVs carrying immunomodulatory factors, anti-inflammatory molecules, myelin repair proteins, or regulatory miRNAs have shown therapeutic effects, reduced neuroinflammation, and improved neurological outcomes in MS models [141–144]. EV-based strategies for modulating immune responses, promoting oligodendrocyte function, and enhancing neural repair hold promise for MS treatment approaches.

In conclusion, preclinical studies utilizing animal models have provided valuable insights into the therapeutic potential of EVs across a wide range of neurodegenerative disorders, including neurodegenerative diseases [140, 145, 146]. These studies have demonstrated the efficacy, safety, and mechanisms of EV-mediated neuroprotection, neurodegeneration, immune modulation, and functional recovery in various CNS conditions. Continued research efforts are needed to optimize EV-based therapies, validate their efficacy in clinical trials, and advance toward personalized medicine approaches for neurodegenerative disorders.

## Future perspectives and conclusions

EVs have gained significant attention as promising therapeutic agents and diagnostic tools for neurodegenerative diseases. Table 2 summarizes ongoing clinical trials utilizing EVs for the treatment of these conditions. Most trials employ EVs derived from MSCs, leveraging their regenerative and anti-inflammatory properties. Common delivery routes include intravenous (iv), intranasal, and localized injections, tailored to the target disease and desired therapeutic efficiency. Although the field of EV-based therapies is still emerging, the number of trials specifically focused on neurodegenerative diseases remains limited. With advancements in emerging technologies, novel therapeutic targets, and personalized medicine approaches, EV research continues to evolve rapidly. Future progress in EV isolation and characterization, the exploration of innovative therapeutic strategies, and the development of personalized EV-based therapies hold immense potential to revolutionize the management of neurodegenerative diseases.

Exploration of novel therapeutic targets and strategies based on EV biology holds immense potential for developing targeted interventions, disease-modifying treatments, and regenerative therapies in neurodegenerative disorders. Future research directions include: 1) EV cargo engineering: Engineering EV cargo, such as miRNAs, siRNAs, small molecules, proteins, and therapeutic peptides, offers precise control over therapeutic payload delivery, target specificity, and therapeutic efficacy [15, 147]. Strategies for loading, modifying, and functionalizing EV cargo enable tailored therapeutic interventions, gene silencing mechanisms, and molecular interactions with disease-specific pathways. 2) EV-directed drug delivery: Utilizing EVs as drug delivery vehicles for neuroprotective agents, neurotrophic factors, gene editing tools, or disease-modifying drugs enhances targeted delivery, sustained release, and therapeutic potency within the CNS [48]. EV-based drug delivery systems overcome BBB limitations, improve bioavailability, and reduce off-target effects, optimizing treatment outcomes and minimizing systemic toxicity. 3) EV-mediated immunomodulation: EV-based immunomodulation strategies modulate immune responses, enhance tissue repair mechanisms, and promote neuroprotection in neurodegenerative diseases [148]. 4) EV-based neuroregeneration: Exploiting EV-mediated neuroregenerative properties, including neural stem cell differentiation, axonal growth promotion, and synaptogenesis enhancement, supports neuronal repair, functional recovery, and neural circuit remodeling in neurodegenerative conditions [15]. EV-based neuroregeneration strategies stimulate endogenous repair mechanisms, enhance neuroplasticity, and restore neuronal connectivity, offering potential for restoring lost functions and improving patient outcomes. 5) EV-mediated neuroprotection: Targeting EV-mediated neuroprotective mechanisms, such as antioxidant activity, mitochondrial support, and anti-apoptotic effects, provides neuroprotection, neuronal survival, and resilience against neurodegenerative insults. EV-based neuroprotective strategies mitigate oxidative stress, enhance cellular viability, and preserve neuronal function, contributing to disease modification and symptom alleviation in neurodegenerative disorders.

The efficiency of EVs as drug carriers is often limited by their biological properties, such as short circulation half-life, low targeting efficiency, and limited drug-loading capacity. To address these challenges, various strategies have been developed to modify and engineer EVs, enhancing their therapeutic potential [149]. One approach is chemical conjugation, where molecules are attached to the surface of EVs using techniques like click chemistry. This method is highly efficient, specific, and biocompatible, enabling the attachment of targeting ligands such as antibodies or peptides for targeted drug delivery [150]. Another strategy is genetic modification, which involves altering the genetic material of EV-producing cells to express specific proteins or peptides on the EV surface. For instance, RVG-modified EVs loaded with therapeutics have shown improved efficacy in disease models, including AD [151, 152], major depressive disorder (MDD) [146] and neuroinflammation [147]. Physical modification methods, such as liposome fusion and membrane permeabilization, have also been employed to enhance EV drug-loading capacity and targeting efficiency [153]. These techniques can be combined with genetic modification, allowing further customization of EV-cell interactions by altering lipid composition or properties [154]. In summary, the modification and engineering of EVs for drug delivery represent a promising area of research with broad therapeutic applications. While current methods each have their strengths and limitations, the development of more efficient and precise techniques will be essential to fully realize the therapeutic potential of EVs.

Overall, future perspectives and directions in EV research for neurodegenerative diseases encompass technological advancements, novel therapeutic targets, and personalized medicine approaches. Leveraging emerging technologies for EV isolation and characterization, exploring new therapeutic strategies based on EV biology, and harnessing the potential for personalized EV-based therapies offer transformative opportunities for improving patient outcomes, disease management, and healthcare advancements in neurodegenerative disorders. Characterizing patient-specific EV profiles, biomarker signatures, and disease-associated EV cargoes enables precision diagnostics, treatment selection, and therapeutic monitoring tailored to individual patient needs. Integrating multi-omics data, imaging biomarkers, and clinical phenotypes facilitates personalized medicine approaches and prognostic assessments in neurodegenerative disorders [15, 155]. Continued research, interdisciplinary collaborations, and translational efforts are essential for realizing the full potential of EV-based interventions in neurodegeneration.

In conclusion, EVs hold tremendous potential as a therapeutic frontier for neurodegenerative diseases, offering novel insights, personalized treatment strategies, and transformative opportunities for improving patient outcomes and advancing neurodegenerative disease treatment paradigms. Continued research efforts, collaborative initiatives, and translational endeavors are essential for unlocking the full therapeutic potential of EV-based therapies and shaping the future of neurodegenerative disease management.

## Figures and Tables

**Table 1.** Preclinical studies of EVs as delivery vehicles in neurodegenerative disorders

Disease	EV source or cargo	Animal models	Effects	References
Alzheimer’s disease	Mesenchymal stem cells (MSCs)-derived EVs	APP/PS1 and icv-STZ mice	Alleviate Aβ-induced iNOS and inflammation	(124, 126)
	Adipose-derived mesenchymal stem cells (ADSCs-derived EVs)	APP/PS1 mice	Reduce Aβ deposition and decrease microglia activation	(125)
	Neural stem cell-derived exosomes	APP/PS1 and 5xFAD mice	Promote mitochondrial biogenesis and restore abnormal protein distribution	(127, 128)
	EVs-mediated delivery of CB2 receptor agonist	APP/PS1 mice	Enhance neuronal regeneration	(123)
Parkinson’s disease	CCR2-enriched mesenchymal stem cell-derived EVs (MSCCCR2 EVs)	MPTP-induced PD mice	Block the infiltration of peripheral inflammatory cells	(129)
	Human umbilical cord mesenchymal stem cell-derived exosomes (HucMSC-EVs)	6-OHDA-induced PD mice	Activate the Wnt/β-catenin pathway and reduce autophagy	(130)
	Human umbilical cord blood-derived mononuclear cells (hUCB-MNCs) enriched with miR-124-3p (miR-124-3p sEVs)	6-OHDA-induced PD mice	Induce neuronal differentiation and protect N27 dopaminergic cells	(131)
	Human neural stem cell-derived EVs	6-OHDA-induced PD mice	Reduce intracellular reactive oxygen species (ROS) and associated apoptotic pathways.	(132)
Huntington’s disease	Young serum-exosomes	R6/2 mice model	Reduce mHtt aggregation protein and apoptotic signaling	(133)
	Human cord blood-derived EVs	3-NP-induced HD rats	Reduce neuroinflammation	(134)
	DNAJB6b-enriched neural stem cell (NSCs)-derived EVs	R6/2 mice model	Reduce mutant HTT aggregation	(135)
	Fibroblast-derived EVs	HD-derived neuron cultures	Increase the density of inhibitory synapses	(136)
Amyotrophic lateral sclerosis	Mesenchymal stroma-/stem-like cells (MSC) -derived EVs	SOD1G93A transgenic primary motor neurons	Antioxidant and anti-apoptotic pathways	(137)
	Adipose-derived stem cell-derived exosomes (ASC-exosomes)	SOD1(G93A) mice	Improve motor performance; protect lumbar motoneurons; and decrease glial activation.	(138)
	Adipose-derived stem cell exosomes	Neuronal cells from G93A ALS mice	Reduce cytosolic SOD1 level	(139)
	Regulatory T Cell-derived EVs	SOD1 mice	Increase survival, and modulate inflammation	(140)
Multiple sclerosis	Oligodendrocyte precursor cell-derived exosomes	Experimental autoimmune encephalomyelitis (EAE) mice	Reduce microgliosis and astrogliosis	(141)
	Amniotic fluid stem cell-derived EVs	EAE mice	Reprogramming inflammatory cDC2s	(142)
	Mesenchymal stem cell-derived EVs containing miR-181a-5p	EAE mice	Inhibit microglial inflammation and pyroptosis through the USP15-mediated RelA/NEK7 axis.	(143)
	Adipose mesenchymal stem cell-derived EVs	EAE mice	Target inflamed lymph nodes	(144)

**Table 2.** Clinical trials investigating the use of extracellular vesicles in the treatment of neurodegenerative diseases.

Disease	Source of EVs	Route of Administration	Dose	Duration	Clinical Trial Phase	Recruitment Status	ClinicalTrial ID
Alzheimer’s Disease	Allogenic Adipose Mesenchymal Stem Cells	Nasal drip	5–20μg	Twice a week for 12 weeks	Phase 1/2	Unknown status	NCT04388982
Multiple neurodegenerative diseases	Human umbilical cord mesenchymal stem cells	Nasal drops	Not specified	Not specified	Phase 1	Not yet recruiting	NCT06607900
Amyotrophic Lateral Sclerosis (ALS)	Human umbilical cord blood mesenchymal stem cells	Nasal drops	Dose-escalation	Twice a week for two weeks	Phase 1/2	Recruiting	NCT06598202
Depression, Anxiety, and Dementias	Healthy, full-term Cesarean section amniotic fluid	Focused ultrasound and intravenous infusion	Not specified	Not specified	Not Applicable	Suspended	NCT04202770

## Abbreviations

AD: Alzheimer’s disease
ALS: amyotrophic lateral sclerosis
Aβ: amyloid-beta
BBB: blood-brain barrier
CNS: central nervous system
ESCRT: endosomal sorting complex required for transport
EVs: extracellular vesicles
HD: Huntington’s disease
mHTT: mutant huntingtin protein
MS: multiple sclerosis
MSCs: mesenchymal stem cells
MVBs: multivesicular bodies
NPCs: neural progenitor cells
PD: Parkinson’s disease
SOD: superoxide dismutase
